# Hydrothermal Synthesis of CaAl-LDH Intercalating with Eugenol and Its Corrosion Protection Performances for Reinforcing Bar

**DOI:** 10.3390/ma16072913

**Published:** 2023-04-06

**Authors:** Ang Liu, Haohua Gu, Yongjuan Geng, Pan Wang, Song Gao, Shaochun Li

**Affiliations:** School of Civil Engineering, Qingdao University of Technology, Qingdao 266520, China; manyi0076@163.com (A.L.);

**Keywords:** CaAl-LDH, hydrothermal synthesis, organic inhibitor, corrosion protection, reinforcing bar

## Abstract

Layered double hydroxides (LDHs) intercalating with a corrosion inhibitor for slowing down the corrosion of a reinforcing bar has attracted considerable attention. However, achieving high-loading capacity of organic inhibitor in LDH with high efficiency and long-term protection characteristics remains an important challenge. In this work, the CaAl-LDH intercalating with eugenol (EG) was synthesized via a continuous hydrothermal method. The prepared LDHs were characterized by SEM, XRD, UV-vis absorption spectra and TGA. Additionally, the corrosion protection performances of LDH-EG for steel bar were studied in detail via the electrochemical method. The results show that the loading amount of EG in LDHs was about 30% and about 80% EG could be released from LDH-EG within 4 h in SCPs containing 3.5% NaCl. The electrochemical test results show that the *R_ct_* value (10^5^~10^6^ Ω · cm^2^) of steel-mortar incorporated with LDH-EG has increased by 3–4 orders of magnitude compared to the specimen without LDHs (10^2^~10^3^ Ω · cm^2^) after 16 dry–wet cycles corrosion test. The significantly improved protection capability is mainly derived from two aspects: one is the filling effect of LDH, which can fill the pores of mortar and improve the impermeability; another reason is that the intercalated EG can slowly diffuse out of the inner structure of LDHs in a controllable way and result in a relatively long-term effect of corrosion inhibition.

## 1. Introduction

Chloride-induced corrosion of reinforcing bars have been regarded as the leading cause of deterioration of reinforced concrete structures in a marine environment [1,2]. The damage of reinforced concrete structures not only causes huge economic losses, but also results in potential safety hazard and environmental consequences. Incorporation of corrosion inhibitor into cementing materials during concrete preparation is an effective and practical measure to delay the corrosion of the reinforcing bar due to the convenient operation and low cost [3,4]. However, the use of corrosion inhibitors still confronts many problems and challenges, such as short duration, low efficiency (migrating corrosion inhibitor), fast decomposition, and high toxicity (e.g., nitrite and benzoate) [4,5]. It is necessary to develop the next-generation corrosion inhibitor with low toxicity, high efficiency, and longer lasting protection.

In recent years, the utilizing of micro-/nano-materials [6,7,8] as a carrier container to achieve corrosion protection with multifunction is considered to be an ingenious and efficient strategy. Furthermore, layered double hydroxides (LDHs) have attracted the interest of researchers due to the unique layered structure and the controllable composition, especially the intrinsic anion exchange function that makes it gradually become a research hotspot in the corrosion protection field [9,10,11]. Ferreira et al. [12,13] have carried out a lot of original work on utilizing LDHs as a nanocontainers for anticorrosion applications, and clarified that LDHs perform a double rule: providing inhibitors to protect the metallic substrates and entrapping aggressive species from the environment. In the field of concrete, LDHs were originally adopted for the loading of a water reducer to control the hydration of cement [14]. Subsequently, LDHs were applied to absorb corrosive ions in concrete. Shui et al. [15] studied the influence of LDHs and its calcinated product on the durability of concrete, they found that the LDHs additive can not only effectively reduce the micro-pores in concrete, but also absorb a large amount of CO_3_^2−^. Yang et al. [16,17] explored the protection performance of LDHs and its derivatives for reinforcement, and suggested that LDHs can effectively prevent the chloride induced corrosion when replacing a mass of 5% of cement by LDHs in bulk mortar or as a coating of reinforcing steel (LDHs to replace 20% mass of cement). Xu et al. [18,19] reported that LDHs intercalated nitrite exhibits good corrosion inhibition for steel bar in simulated concrete pore solution containing Cl^−^ or SO_4_^2−^ due to the anion exchange between NO_2_^−^ and corrosive ions on the Mg-Al LDHs. Mir et al. [20] summarized the influence of different types of LDHs on cement hydration based on previous studies, and found that ZnAl LDHs will dissolve Zn^2+^ from LDHs laminate in the highly alkaline environment, and the dissolved Zn^2+^ can complex with Ca^2+^ and OH^−^, thus delaying or even hindering cement hydration. On the contrary, CaAl, MgAl, and LiAl LDHs will promote the hydration of cement, in particular, CaAl LDHs will promote the formation of hydrated calcium silicate (C-S-H), which is more appropriate to be used as an admixture for corrosion protection of reinforced concrete.

Although numerous studies show that LDHs has great potentialities as a corrosion inhibitor delivery carrier to inhibit the corrosion of steel bar, the loading amount of corrosion inhibitor (especially the organic corrosion inhibitor with large molecular weight) in LDHs, and the simple and efficient synthesis method remain an important challenge, which are important for the long-term corrosion protection and practical application in concrete [11]. Eugenol (EG), the extracts of Cinnamomum verum leaf, which are cost-effective, renewable, and bio-compatible sources for green inhibitors and can highly diminish the metal corrosion [21,22]. Therefore, we synthesized the CaAl-LDH intercalating with organic corrosion inhibitor EG via continuous hydrothermal method to achieve high loading amount and long-term corrosion protection properties. The anion release kinetics and loading capacity of LDHs was studied. Additionally, the corrosion protection performances of LDHs loaded with EG for steel bar in solution and mortar were studied in detail by electrochemical method. Additionally, the mechanism of the significantly improved protection performance was discussed in detail.

## 2. Materials and Methods

### 2.1. Materials

A reinforcing bar is mild steel and the chemical composition is: 0.16 C, 0.53 Mn, 0.30 Si, <0.045 P, <0.055 S, 0.3 Ni, 0.3 Cu (wt.%), and Fe balance. Portland cement (CEM I 42.5), river sand (fineness modulus 2.5) and deionized water were used for preparing mortar. The above materials were purchased from Qingdao Dongshengdi Trading Co., Ltd. (Qingdao, China) Eugenol (EG) was selected as green organic corrosion inhibitor. All chemicals used for material synthesis and characterization are analytical reagents and purchased from Sinopharm Chemical Reagent Co., (Shanghai, China).

### 2.2. Hydrothermal Synthesis of CaAl-LDH Intercalated with EG

CaAl-LDH intercalated with EG was synthesized via a continuous hydrothermal method. Briefly, a mixed solution A, containing 0.5 M Ca(NO_3_)_2_ · 4H_2_O and 0.25 M Al(NO_3_)_3_ · 9H_2_O, and a mixed solution B, containing 1.5 M NaNO_3_ and 2 M NaOH, were dropwise added into a five-necked flask. The dripping speed of solution A and B was controlled by a peristaltic pump according to the pH value (maintained at 11 ± 0.5) of the mixed solution. Additionally, the mixed solution kept a continuous agitating at 70 °C to achieve the uniform synthesis of CaAl-LDH. After a period of operation (the operation time was determined according to the volume of five-necked flask), the CaAl-LDH suspension was directly introduced into 0.1 M deprotonated EG aqueous solution (Equimolar amounts of EG and NaOH mixed solution) under vigorous stirring for 24 h at 70 °C. Finally, the product (LDH-EG) was collected by centrifugation, washed three times with deionized water and absolute ethanol, respectively, and dried at 45 °C for 2 h. The above synthesis process was operated continually under nitrogen atmosphere and all the solutions were made by using double distilled water.

### 2.3. Fabrication of LDHs Incorporated Mortar with Embedded Reinforcing Bar

**Figure 1:**
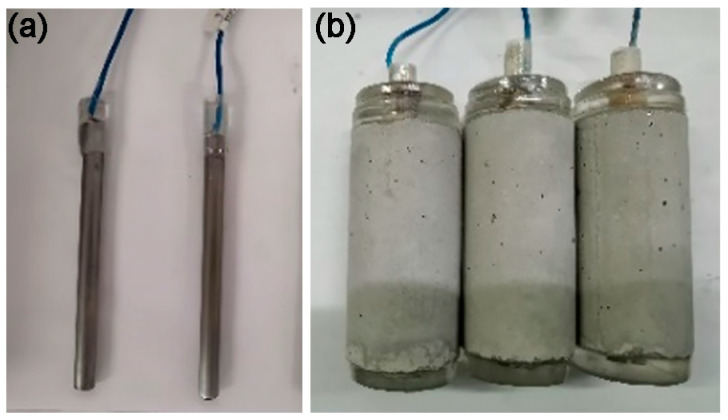
(**a**) the reinforcing bar welded with copper wire; (**b**) the LDHs incorporated mortar specimens with embedded reinforcing bar.

The reinforcing bar was cut into pieces with a diameter of 10 mm and length of 150 mm, a copper wire was welded on one end of reinforcing bar, and the welding spot was sealed up with epoxy resin. The mortar was fabricated with a water-cement mass ratio of 0.5 and sand-cement mass ratio of 3, and the mixing amount of LDHs (to replace cement) is 3% of the cement. The mortar with embedded reinforcing bar was fabricated by using a cylindrical mold, and the final product was shown in Figure 1. The cylindrical in shape of reinforced mortar samples aims to provide a same diffusion path for chloridion to reinforcing bar.

### 2.4. Materials Characterization

The morphological features of the obtained LDHs were characterized by field emission scanning electron microscope (SEM, Carl Zeiss, Oberkochen, Germany), and the structural feature of LDHs powders were studied by X-ray diffraction, XRD, Rigaku Ultima IV, Cu Kα radiation, with a scanning rate of 10° min^−1^ range from 5 to 80° 2*θ*. The chemical components of EG and LDHs were characterized by Fourier transform infrared spectroscopy (FTIR, Nicolet iS10 spectrometer, Thermo Fisher Scientific, Waltham, MA, USA). The release kinetics and loading capacity of LDHs were determined by using UV-vis absorption spectra (Hitachi U-2900 spectrophotometer, Hitachi Limited, Tokyo, Japan, in the range of 200–400 nm) and thermogravimetric analysis (TGA: SDTQ600 comprehensive thermal analyzer, TA Instruments, New Castle, DE, USA, the test was conducted from room temperature to 800 °C with a heating rate of 10 °C min^−1^ under nitrogen flow) according to our previous research [23]. In the case of UV-vis absorption spectra experiment, 100 mg of LDHs (with a dosage of 2 g/L) was dispersed into 50 mL simulated concrete pore solution (SCPs) containing 3.5 wt.% NaCl under continuous stirring. The mixed solution was stirred for a fixed time (up to 48 h), then centrifuged, and a very small amount of the supernatant was taken for analysis. In addition, a UV-vis absorption spectra calibration curve of pure EG in SCPs with NaCl solution was obtained for quantitative analysis.

### 2.5. Corrosion Protection Performances Evaluation

The corrosion protection performances of LDH-EG were explored by electrochemical impedance spectroscopy (EIS). First, the corrosion inhibition behavior of pure EG and LDH-EG powders for mild steel was tested at different immersion times in SCPs containing 3.5 wt.% NaCl. The added amount of EG and LDH-EG in the test solution were 10 mmol/L and 1 g/L, respectively. Finally, the corrosion protection performances of LDH-EG for steel bar were further studied by using the mortar-reinforcing bar specimen (Figure 1b) in 3.5% NaCl solution during 16 dry-wet cycles. The EIS was conducted on Autolab PGSTAT302 N workstation with a conventional three-electrode cell, where the mild steel samples were used as a working electrode, a platinum plate was selected as counter electrode, and a saturated calomel electrode served as reference electrode, and the frequency range is from 1 MHz to 10 mHz and applied amplitude is 5 mV. All EIS test was repeated three times to ensure the reproducibility and the EIS data was further analyzed through Zview 3.1 software.

## 3. Results and Discussion

### 3.1. Characterization of LDHs

**Figure 2:**
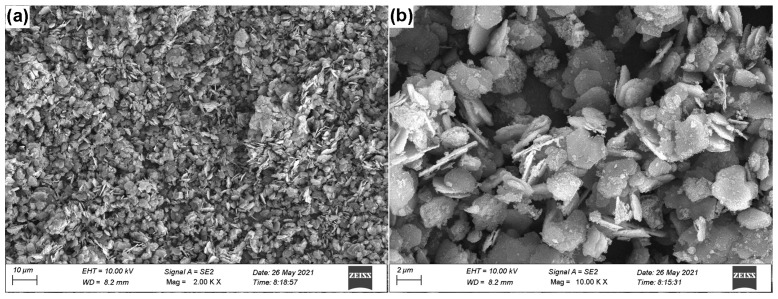
SEM images of: (**a**) the synthesized CaAl LDH–NO_3_^−^; (**b**) the enlarged SEM images of (**a**).

**Figure 3:**
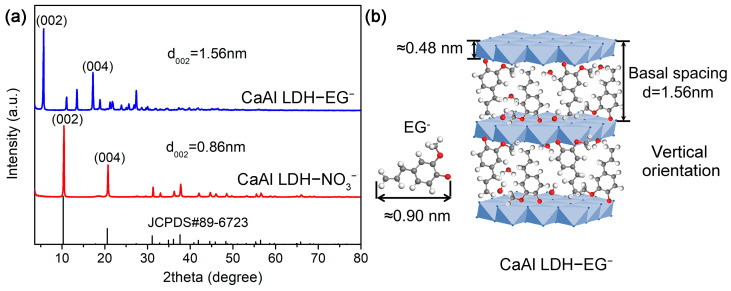
XRD patterns of: (**a**) CaAl LDH–NO_3_^−^ and CaAl LDH–EG^−^; (**b**) The orientation diagram of EG^−^ in the interlayer space of CaAl LDH.

**Figure 4:**
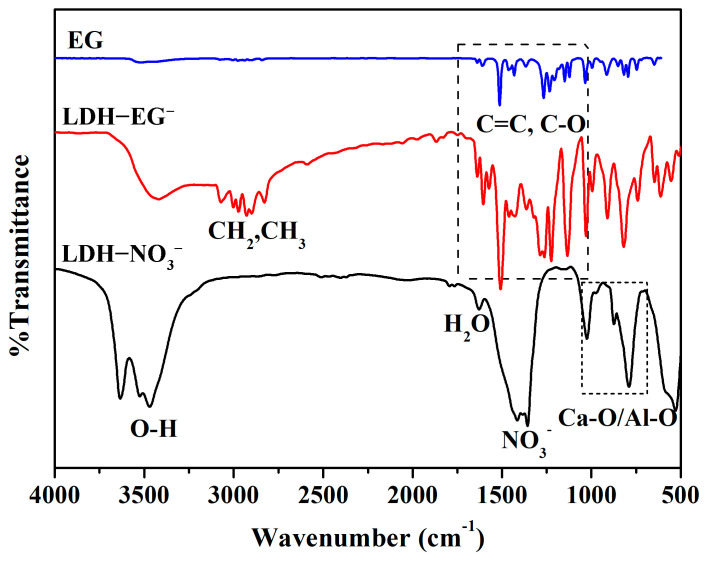
FTIR spectra of EG and prepared CaAl LDHs.

Figure 2 shows the SEM images of the synthesized CaAl LDH-NO_3_^−^. It can be clearly seen that the LDHs exhibit pseudo-hexagonal platelet structure and have even size distribution (with a platelet size of about 2 μm), indicating that the prepared LDHs have a good crystallinity and dispersion [11,20]. The XRD results were shown in Figure 3. We can find that the XRD patterns of CaAl-LDH-NO_3_^−^ (Figure 3a) exhibits typical diffraction peaks of (002) and (004), and the calculated basal spacing (d_002_) is 0.86 nm, which are in good agreement with the powder diffraction standards (JCPDS#89-6723) [24,25]. After intercalating with corrosion inhibitor EG, the typical diffraction peaks of (002) and (004) are shifted to the low 2*θ* value, and the calculated basal spacing (d_002_) is 1.56 nm, indicating that the successful loading of EG anions. This result is slightly bigger than the theoretical calculations (about 1.38 nm) of basal spacing, which is equal to sum of the thickness of monolayer LDH (about 0.48 nm) [12,26] and the length of EG anions (about 0.9 nm), demonstrating the vertical alignment of EG anions in the interlayer of LDHs. It is noteworthy that a few sub-peaks can be observed at 2*θ* = 11°, 13°, 18~26°, respectively; this is probably the insoluble salts of EG, calcium, and aluminum hydroxides, respectively [27]. The SEM and XRD results demonstrate the high-quality synthesis of LDH-EG through the above method. The FT-IR results further verified the synthesis of LDH-EG, as shown in Figure 4. In the spectrum of CaAl LDH-NO_3_^−^, the absorption peaks at about 3500 cm^−1^ are due to the O-H stretching modes of hydroxyl group and interlayer H_2_O, and the weak absorption peak at about 1600 cm^−1^ can be linked to H_2_O molecules [23,24]. The peaks at about 1380 are assigned to the stretching vibrations of N-O in nitrate [28]. Additionally, the peaks below 1000 cm^−1^ can be attributed to metal-oxygen (Ca-O, Al-O) stretching and bending modes [24,28]. After intercalating with EG, the spectrum shows obvious characteristic peaks of EG. The peaks at about 3000 cm^−1^ and 2900 cm^−1^ are attributed to -CH_3_ and -CH_2_, and a series of vibration peaks between 1600 cm^−1^ and 1000 cm^−1^ are assigned to C=C and C-O stretching [29,30]. The above results clearly verified the successful synthesis of LDHs intercalating with EG.

### 3.2. The Release Kinetics and Loading Capacity of LDHs

**Figure 5:**
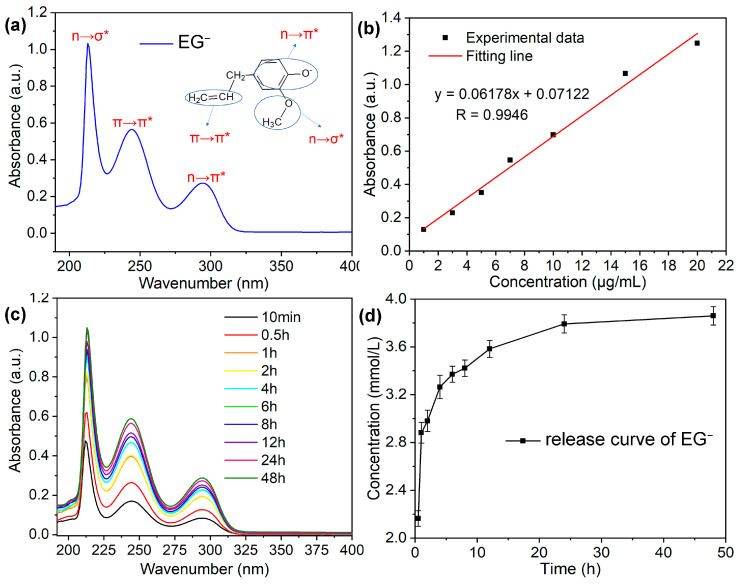
(**a**) UV–vis absorption spectra of pure EG^−^ in SCPs solution containing 3.5 wt.% NaCl; (**b**) The plot of UV–vis calibration curve of pure EG^−^ in SCPs with NaCl solution obtained by using linear fitting method; (**c**) UV–vis absorption spectra of EG^−^ released from LDH into SCPs with NaCl solutions within 48 h; (**d**) EG^−^ release kinetics from LDH–EG^−^ into SCPs with NaCl solutions within 48 h.

A UV-vis absorption spectra experiment was performed to determine the release kinetics of EG anions from the LDHs interlayer, as shown in Figure 5. Figure 5a shows the typical UV-vis absorption spectra of pure EG anions in SCPs with NaCl solutions. The spectra clearly presents 3 typical absorption peaks, which can be assigned to the n–σ* (215 nm), π–π* (245 nm), and n–π* (295 nm) absorption peaks of EG. The UV-vis absorption spectra calibration curve of pure EG was drawn according to absorbance value at the wavelength of 215 nm, as shown in Figure 5b. Figure 5c shows the UV-vis absorption spectra of EG anions released from LDHs into SCPs with NaCl solutions variation with time, the spectra are similar to the absorption spectra of pure EG. Therefore, the EG release kinetics curve from LDHs into SCPs with NaCl solutions can be obtained from the above results, as shown in Figure 4d. The release process can be divided into 3 phases: the rapid release phase (0~4 h, about 80% of EG was released), the slow-release phase (5~24 h), and the releasing balance phase (24~48 h), which is similar to that of reference [12,23]. The results show that the prepared LDH-EG has the rapid Cl–stimuli responsive release performance. Additionally, the maximum loading amount of EG in LDHs can be calculated according to the following equation:

3.8 mmol/L (C_EG_) × 1 L ÷ 1000 × 164 g/mol (M_EG_) ÷ 2 g (m_LDHs_) × 100% = 31% (1)

This result is significantly higher than that of the previous research [12,31], where the loading amount of phosphate, MBT, and QAin in LDHs is about 1.2%, 19%, and 25%, respectively, according to the calculation Formula (1).

**Figure 6:**
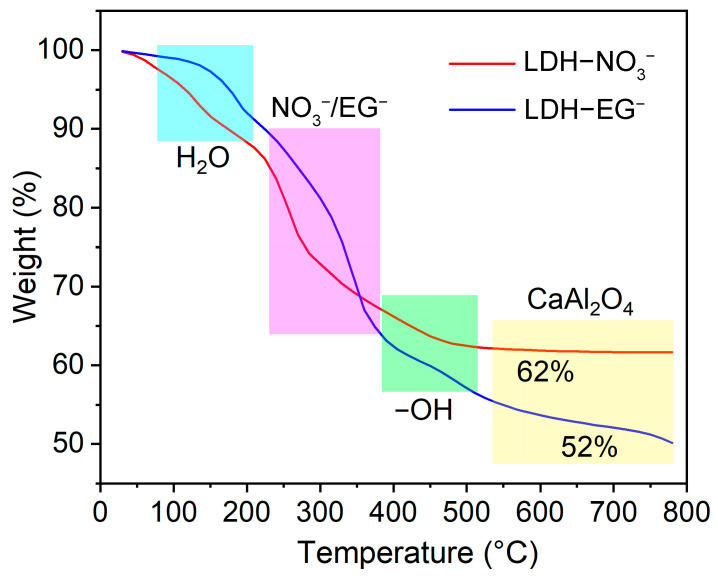
TG profiles for CaAl LDH–NO_3_^−^ and CaAl LDH–EG^−^.

The loading capacity of the prepared LDHs was further determined by TGA, as shown in Figure 6. The TG curves show that the thermal decomposition process of LDHs can be divided into four stages, which is similar to other types of LDHs (ZnAl, MgAl, and NiAl LDHs) [32,33,34]. The first stage range from room temperature to about 150 °C can be attributed to the thermal decomposition of adsorbed and intercalated water molecule; the second stage range from 150 °C to 380 °C corresponding to the loaded species (NO_3_^−^, EG^−^, etc.) in the interlayer of LDHs [23,32]; the other two stages within the range of 380 °C to 550 °C and 550 °C to 800 °C can be assigned to the thermal decomposition of -OH and the formation metallic oxide (CaAl_2_O_4_) [34]. Additionally, the XRD and FTIR results clearly show that the EG has successfully substitutes NO_3_^−^ in the interlayer of LDHs. Therefore, we can estimate the content of loaded EG from the second weight loss stage, which shows that the intercalated EG is about 30% (the weight loss from 90% to about 60%). The result is close to the result of UV-vis absorption spectra experiment, verifying the high loading amount of organic corrosion inhibitor in the obtained LDHs.

### 3.3. Corrosion Inhibition Performances of LDHs for Mild Steel in SCPs with 3.5% NaCl

**Figure 7:**
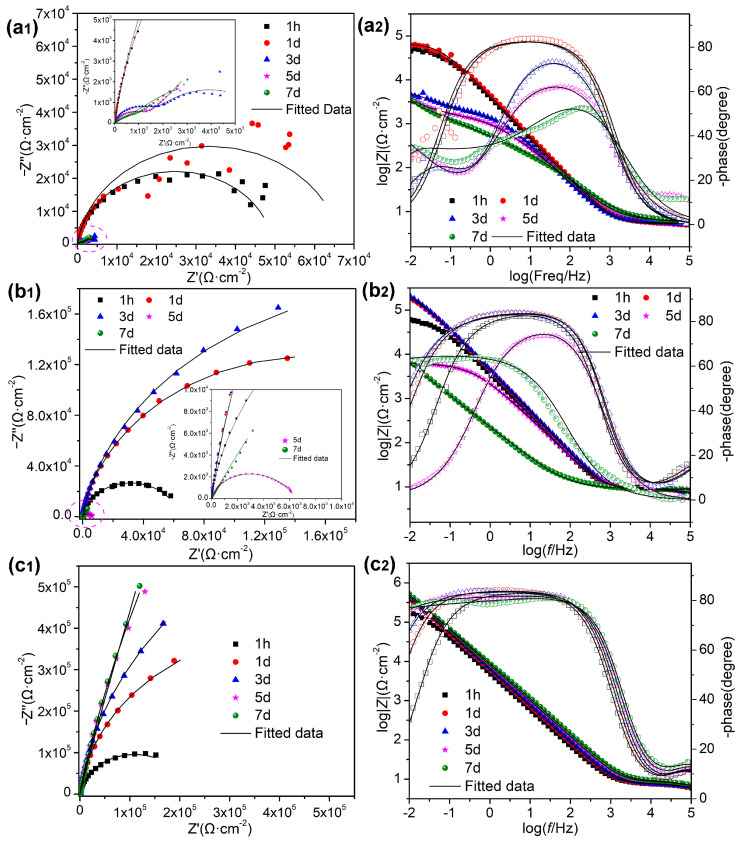
Nyquist (**left**) and Bode (**right**) plots of the mild steel electrodes at different immersion time in SCPs solution with 3.5 wt.% NaCl solution: (**a_1_**,**a_2_**) without EG, (**b_1_**,**b_2_**) containing 10 mmol/L EG, (**c_1_**,**c_2_**) containing 1 g/L LDH–EG.

The corrosion inhibition performances of LDHs for mild steel in SCPs with 3.5% NaCl were investigated by EIS. Figure 7 displays Nyquist and Bode plots of the mild steel electrodes at different immersion times in test solutions with and without LDHs. In the solution without EG and LDH-EG, the Nyquist plot presents a large diameter of capacitive loop after 1 h and 1 d of immersion, indicating a passivation film was formed on the surface of mild steel and inhibits the corrosion [18,35]. It is noteworthy that the Nyquist and Bode plots have some abnormal volatile points at low frequency region at the immersion time of 1 d, this phenomenon can be attributed to the competition adsorption between Cl^−^ and OH^−^ on the surface of mild steel, and the increased capacitive loop suggests OH^−^ has occupied the advantage [36,37]. However, there appears a new time constant after 3 d of immersion, and the diameter of capacitive loop decreased significantly, revealing that Cl^−^ has taken advantage in the competition adsorption and caused steel bars to lose passivation [36]. After adding EG to the solution, the diameter of capacitive loop increased gradually with the immersion time in the first 3 d; additionally, the bode curves show that the impedance value (at 0.01 Hz) has increased about one order of magnitude after immersion of 3 d and the phase angle has also increased in low-frequency region (increases from 15° to 50° at 0.01 Hz), indicating that the low-frequency relaxation process becomes more obvious. These phenomena can be attributed to the continuously adsorbed corrosion inhibitor film on the surface of mild steel, which suppressed the attack of Cl^−^ [38,39,40]. However, the obviously reduced capacitive loop in the later soaking time shows that Cl^−^ has damaged the protective film. In contrast, the Nyquist plots became more bigger and more vertical and the phase angles are closer to −90° during the whole test period after incorporation with LDH-EG, demonstrating that LDH-EG exhibits excellent corrosion inhibition performance for mild steel under Cl^−^ attack [19].

**Figure 8:**
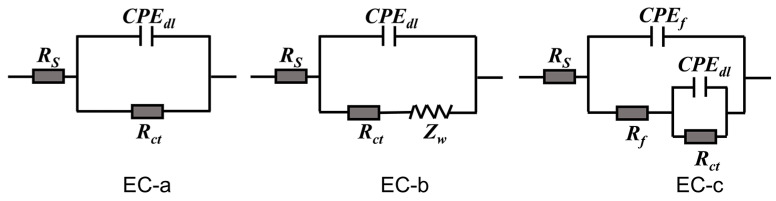
Equivalent electric circuits used for modeling the EIS results of Figure 7.

**Figure 9:**
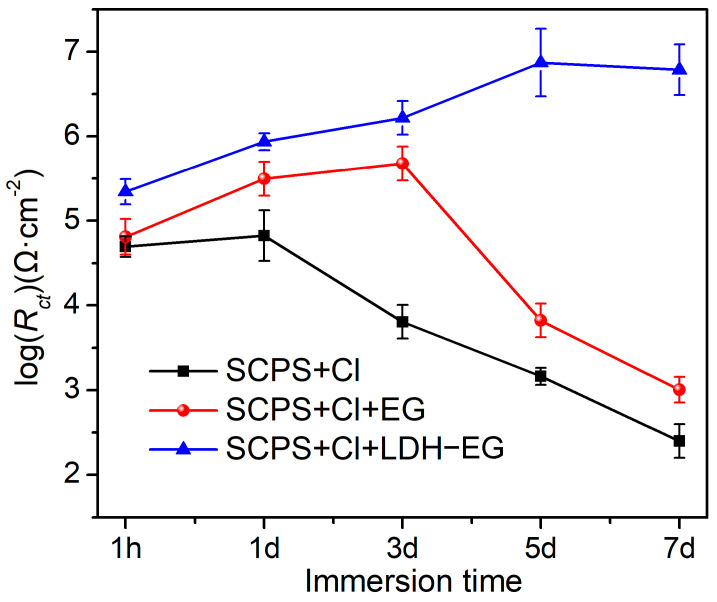
The time-dependent behavior of *R_ct_*.

In order to further interpret the corrosion behavior of mild steel in different solutions, we have proposed three equivalent electric circuit (EC, as shown in Figure 8) models to fitted the EIS data, the corresponding EC and fitting parameters of each EIS plots are listed in Table 1. In the presented EC, *R_s_* is the solution resistance, *CPE_f_* and *R_f_* represent film capacitance and film resistance, respectively, *CPE_dl_* and *R_ct_* corresponding to the electric double-layer capacitor and charge transfer resistance, respectively, and the value of *R_ct_* is inversely proportional to the corrosion rate of mild steel [41]. Figure 9 shows the time–dependent behavior of *R_ct_* value of different experimental subjects. The final parameter *Z_w_* is used to describe the diffusion behavior (finite element diffusion).

From the fitted results, it can be clearly seen that the *R_ct_* value of control group exhibits a decreased trend with immersion time, particularly after 1 day, showing a sharp decline, Additionally, there even presented a diffusion resistance. These phenomena indicate that Cl^−^ can penetrate the passivation film on the surface of mild steel and cause rapid corrosion of mild steel in SCPs. Additionally, the diffusion behavior can be attributed to the accumulated corrosion products restraining the diffusion of corrosive medium. In contrast, the mild steel in the solution with EG presented a film resistance, which is due to the adsorbed corrosion inhibitor layer. Furthermore, the maximum value of *R_ct_* reached to 4.8 × 10^6^ Ω·cm^2^, which has increased by two orders of magnitude compared to the control group. However, the *R_ct_* value shows a trend of increase before decrease, the turning point appeared at the immersion time of 3 d and, accompanied with the disappeared film resistance, suggests that although the organic corrosion inhibitor EG can improve the corrosion resistance of mild steel, but it cannot maintain the protection effect for a long time. In contrast, the *R_ct_* of specimen in the solution with LDH-EG presents the trend of first increase and then tends to be steady, and the maximum value of *R_ct_* is 7.4 × 10^6^ Ω · cm^2^. In addition, the adsorbed corrosion inhibitor film always appears in the immersion period. The above results from this study are in agreement with the conclusion of previous works [10,18,19,23]: the incorporation of LDHs provided a highly efficient and long-term protection performance due to the continuously released corrosion inhibitor and captured Cl^−^.

### 3.4. Corrosion Resistance of LDHs Incorporated Mortar with Embedded Reinforcing Bar

**Figure 10:**
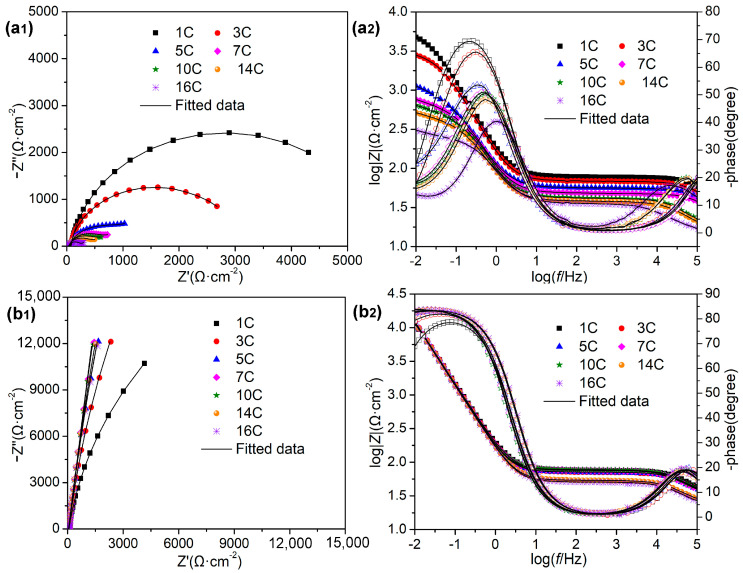
Nyquist (**left**) and Body (**right**) plots of the steel-mortar electrodes at different dry wet cyclic number immersed in 3.5 wt.% NaCl solution: (**a1**,**a2**) without LDH, (**b1**,**b2**) incorporated with 1 wt.% LDH–EG in the mortar.

To further verify the superior corrosion protection performances of LDH-EG for reinforcing bar, the EIS test was also conducted on the specimens of LDHs incorporated mortar with embedded reinforcing bar (steel-mortar electrodes). Figure 10 shows the Nyquist and Body plots of the steel-mortar electrodes with and without LDH-EG at different dry-wet cyclic number immersed in 3.5 wt.% NaCl solution. In general, a large diameter of capacitive loop and high impedance value at 0.01 Hz indicate good corrosion resistance [42]. We can clearly find that the diameter of capacitive loop of the steel-mortar electrodes without LDHs was decreasing persistently with increasing the dry wet cyclic number, indicating the accelerated corrosion rate of steel bar under Cl^−^ attack. In contrast, after incorporation of LDH-EG in the mortar, the Nyquist plots of steel bar shows the trend of first increase, then tends to be steady, and the plots become closer to the imaginary part. Furthermore, the Bode plots of each test were nearly overlapped and the phase angle is close to −90° in the low frequency region, suggesting the superior corrosion resistance of steel bar under the protection of LDH-EG [35,41,42].

**Figure 11:**
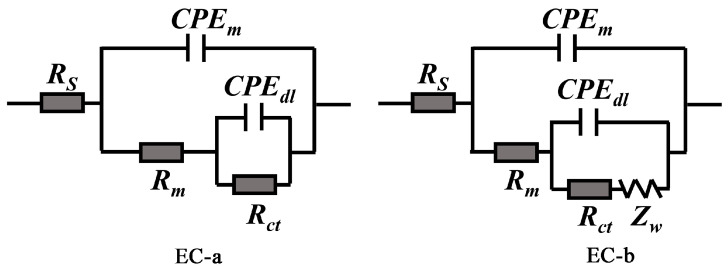
Equivalent electric circuits used for modeling the EIS results of Figure 10.

Two equivalent electric circuit models were proposed to further interpret the corrosion behavior of steel bar, as shown in Figure 10, and the fitted parameters for EIS spectrum were listed in Table 2. In the equivalent electric circuits, the emerging parameters of *CPE_m_* and *R_m_* represent the capacitance and resistance of mortar layer, respectively [42,43]. The high-frequency relaxation process is attributed to the barrier role of mortar layer, which is related to the impermeability of mortar to corrosive media, while the low-frequency time constant is ascribed to the undergoing corrosion activity of mild steel [31]. Figure 11 displays the time-dependent behavior of *R_m_* and *R_ct_* over cycle period. According to above results, the *R_m_* and *R_ct_* of the steel-mortar electrodes without LDHs were decreased continuously over time, and *R_ct_* value is 190 Ω · cm^2^ after16 dry-wet cycles, which is close to the resistance value of bare steel immersed in SCPs solution with 3.5 wt.% NaCl for 7 d (Section 3.3). This is because the Cl^−^ was continuously permeated into the mortar layer and reached to the surface of steel bar, then damaged the passivation film, resulting the aggravated corrosion of steel bar substrate [42]. Furthermore, the diffusion feature occurred in the low frequency region after 5 dry wet cycles, which can be attributed to the accumulated corrosion products on steel bar inhibited the diffusion process and slowed down the corrosion reaction [44,45]. In the case of steel-mortar electrodes incorporated with LDH-EG, the *R_m_* increased slightly during the first 10 cycles, and the *R_m_* value (about 45 Ω · cm^2^) has increased by 80% compared to the control group (about 25 Ω · cm^2^). This result can be attributed to the filling effect of LDHs for mortar [11,24] and the capture action to Cl^−^ of LDHs [13]. The later reduced *R_m_* value could be attributed to the consumption of LDHs. Additionally, the *R_ct_* value increased during the first 10 cycles, suggesting that the released EG improved the corrosion resistance of steel bar. The slight decreased *R_ct_* value after 10 cycles could also be attributed to the consumption of LDHs and EG. It is worth emphasizing that the *R_ct_* value (10^5^~10^6^ Ω · cm^2^) of steel-mortar electrodes with LDH-EG has increased by 3–4 orders of magnitude compared to the specimen without LDHs (10^2^~10^3^ Ω · cm^2^), confirming the significantly enhanced corrosion protection performance of LDH-EG for steel bar.

Furthermore, we have conducted a comparative study of corrosion protection effect of some different LDHs/inhibitors on reinforcing bars [35,46,47,48], as shown in Table 3, and the inhibition efficiency (*η*) is calculated by the following equation [23,49]:

(2) $$\eta \%=\frac{R_{ct}-R_{ct}^{0}}{R_{ct}}\times 100\%$$

where *R_ct_* and *R*^0^*_ct_* are the charge transform resistance of carbon steel in test media with and without of LDHs/inhibitors, respectively. It can be clearly seen that the inhibition efficiency of LDH-NO_2_^−^ is about 99.8%, indicating the superior corrosion inhibition performance of this conventional inorganic inhibitor (NO_2_^−^). However, the high dosage and toxicity of NO_2_^−^ limits its application. The migrating corrosion inhibitor (1,6-hexamethylenediamine, with a dosage of 10 wt.%) shows an inhibition efficiency of about 84.5 and the LDH intercalated organic inhibitor (phthalates) shows a lower inhibition efficiency (70.6). This may be attributed to the low loading capacity of inhibitors in LDHs. In contrast, the LDH-eugenol synthesized in this paper exhibits excellent corrosion inhibition performance in a low dosage and the inhibition efficiency exceeds 99% in both simulated concrete pore solution and mortar specimens.

**Figure 12:**
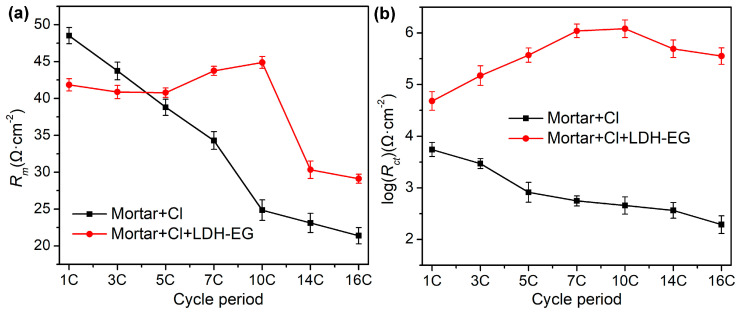
The time-dependent behavior of *R_m_* and *R_ct_*: (**a**) *R_m_* variation with time of steel-mortar electrodes, (**b**) *R_ct_* variation with time of steel-mortar electrodes.

From the above results, we can clearly find that the corrosion resistance of mild steel is significantly improved after incorporation of CaAl-LDH-EG in the corrosive media. The corrosion protection mechanism can be explained based on the results and recent research. In sum, the corrosion inhibitor (EG) can be adsorbed on steel via electrostatic interactions to form a protective layer until an adsorption equilibrium was reached [33,34]. However, the protective layer will be damaged and lose protective capability under constant attack by Cl^−^. This phenomenon can be seen in Figure 7b, where the impedance value has fallen sharply after 3 d of immersion. In contrast, LDH-EG is responsive to the anion (Cl^−^) and releasing inhibiting species (EG) from interlayer galleries based on ion exchange [13,49], leading to a decreasing content of free chlorides in corrosive media. Furthermore, the EG release kinetics curve (Figure 5d) confirms that the release process was governed by a dynamic equilibrium and exchange isotherm [12,50]. Of course, an XRD analysis can further prove the anion exchange process [12,23]. Simultaneously, the released EG can continuously adsorb on steel to physically block the steel surface from being corroded [50]. The sustain released corrosion inhibitor and entrapment of active Cl ions by LDHs in corrosive media synchronous increase the corrosion resistance of steel over long durations [42,51]. The ever-increasing *R_ct_* value (Figure 9 and Figure 12) during the corrosion test can well verified the theoretical analysis. In addition, the filling effect of LDHs for mortar can also decrease the permeability of corrosive media [17,35].

## 4. Conclusions

In summary, we have synthesized CaAl-LDH intercalated with organic corrosion inhibitor (EG) via a continuous hydrothermal method to achieve high loading amount and long-term protection properties. The XRD result verified the intercalation of EG anions into the gallery of the LDH structure (with a basal spacing of 1.56 nm). The release kinetics curve shows that about 30% EG was determined in the interlayer of prepared LDHs. Furthermore, about 80% EG could be released from LDH-EG within 4 h in SCPs containing chloridion, indicating the rapid Cl–stimuli responsive release performance of LDH-EG. The electrochemical experiments shows that the mortar resistance (about 45 Ω·cm^2^) and charge transfer resistance (about 10^6^ Ω·cm^2^) has increased by 80% and 4 orders of magnitude, respectively, after incorporation with LDH-EG into mortar during 16 dry-wet cycles immersion by 3.5% NaCl solution. The significantly improved protection performance is because the filling effect of LDHs can decrease the permeability of corrosive media in mortar and the sustain released EG can provide long term protection. The superior corrosion protection performance of LDH-EG demonstrates its great potential for anticorrosion application in reinforced concrete.

## Figures and Tables

**Table 1:** Fitted parameters for EIS spectrum depicted in Figure 6.

Specimens		*R_s_* (Ω cm^2^)	*CPE_f_* (10^−5^ S s^n^ cm^−2^)	*n_f_*	*R_f_* (Ω cm^2^)	*CPE_dl_* (10^−5^ S s^n^ cm^−2^)	*n_dl_*	*R_ct_* (10^3^ Ω cm^2^)	*W_R_* (10^3^ Ω cm^2^)
SCPS + Cl	1 h	5.6 ± 0.1	–	–	–	4.7 ± 0.03	0.9 ± 0.01	49.5 ± 0.58	–
	1 d	5.3 ± 0.1	–	–	–	4.3 ± 0.08	0.9 ± 0.01	66.7 ± 2.13	–
	3 d	5.4 ± 0.1	–	–	–	8.1 ± 0.16	0.8 ± 0.01	6.4 ± 0.37	–
	5 d	6.1 ± 0.1	–	–	–	14.5 ± 0.53	0.8 ± 0.01	1.5 ± 0.07	4.3 ± 0.16
	7 d	5.8 ± 0.1	–	–	–	17.4 ± 1.16	0.7 ± 0.01	0.25 ± 0.05	5.1 ± 0.37
SCPS + Cl + EG	1 h	3.1 ± 0.1	1.2 ± 0.11	0.7 ± 0.01	6.7 ± 0.7	4.2 ± 0.10	0.9 ± 0.01	64.6 ± 0.82	–
	1 d	4.2 ± 0.1	0.86 ± 0.07	0.7 ± 0.02	6.4 ± 0.3	3.5 ± 0.11	0.9 ± 0.01	313.9 ± 7.52	–
	3 d	3.2 ± 0.1	0.76 ± 0.09	0.6 ± 0.03	9.0 ± 0.05	3.5 ± 0.09	0.9 ± 0.01	475.7 ± 20.1	–
	5 d	8.7 ± 0.1	–	–	–	8.1 ± 0.08	0.9 ± 0.01	6.1 ± 0.04	–
	7 d	3.8 ± 0.1	–	–	–	21.8 ± 0.54	0.8 ± 0.05	1.1 ± 0.12	43 ± 0.86
SCPS + Cl + LDH-EG	1 h	4.8 ± 0.1	0.78 ± 0.05	0.8 ± 0.01	3.2 ± 0.07	2.9 ± 0.05	0.9 ± 0.01	220 ± 1.60	–
	1 d	5.1 ± 0.1	0.29 ± 0.03	0.9 ± 0.02	2.9 ± 0.11	2.7 ± 0.07	0.9 ± 0.01	866 ± 9.67	–
	3 d	5.0 ± 0.1	0.39 ± 0.04	0.9 ± 0.01	3.1 ± 0.07	2.3 ± 0.04	0.9 ± 0.01	1665 ± 33.67	–
	5 d	5.2 ± 0.1	0.68 ± 0.05	0.8 ± 0.01	4.1 ± 0.14	1.6 ± 0.05	0.9 ± 0.01	7425 ± 95.72	–
	7 d	5.3 ± 0.1	0.95 ± 0.06	0.8 ± 0.01	4.8 ± 0.14	2.2 ± 0.02	0.9 ± 0.01	6118 ± 129.9	–

**Table 2:** Fitted parameters for EIS spectrum depicted in Figure 9.

Specimens		*R_s_* (Ω cm^2^)	*CPE_m_* (10^−7^ S s^n^ cm^−2^)	*n_m_*	*R_m_* (Ω cm^2^)	*C_dl_* (10^−3^ S s^n^ cm^−2^)	*n_dl_*	*R_ct_* (10^3^ Ω cm^2^)	*W_R_* (10^3^ Ω cm^2^)
M + Cl	1 C	29.1 ± 1.1	2.4 ± 0.15	0.8 ± 0.01	48.5 ± 1.1	1.1 ± 0.01	0.9 ± 0.01	5.5 ± 0.01	–
	3 C	25.1 ± 1.2	2.8 ± 0.23	0.8 ± 0.01	43.7 ± 1.2	1.3 ± 0.02	0.9 ± 0.01	3.0 ± 0.01	–
	5 C	17.7 ± 1.1	2.5 ± 0.18	0.8 ± 0.01	38.8 ± 1.1	2.2 ± 0.01	0.9 ± 0.01	0.8 ± 0.02	4.7 ± 0.28
	7 C	14.1 ± 1.1	3.2 ± 0.25	0.8 ± 0.01	34.3 ± 1.1	2.2 ± 0.01	0.9 ± 0.01	0.56 ± 0.01	1.3 ± 0.07
	10 C	16.9 ± 0.1	13.0 ± 0.06	0.8 ± 0.01	24.9 ± 0.2	2.4 ± 0.01	0.9 ± 0.01	0.45 ± 0.01	3.9 ± 0.16
	14 C	15.2 ± 0.1	19.2 ± 0.01	0.8 ± 0.01	23.1 ± 0.1	2.7 ± 0.01	0.9 ± 0.01	0.37 ± 0.01	2.1 ± 0.06
	16 C	14.4 ± 0.1	81.1 ± 3.46	0.8 ± 0.01	21.4 ± 0.1	2.3 ± 0.01	0.9 ± 0.01	0.19 ± 0.01	2.4 ± 0.11
M + Cl + LDH-EG	1 C	33.7 ± 0.4	4.2 ± 0.33	0.9 ± 0.01	41.9 ± 0.4	1.0 ± 0.01	0.9 ± 0.01	47.9 ± 0.88	–
	3 C	30.6 ± 0.4	5.9 ± 0.50	0.9 ± 0.01	41.1 ± 0.4	1.1 ± 0.01	0.9 ± 0.01	149.3 ± 8.94	–
	5 C	30.7 ± 0.3	5.7 ± 0.36	0.9 ± 0.01	40.8 ± 0.3	1.1 ± 0.01	0.9 ± 0.01	371.7 ± 34.3	–
	7 C	32.5 ± 0.3	6.7 ± 0.42	0.9 ± 0.01	43.8 ± 0.3	1.1 ± 0.01	0.9 ± 0.01	1092 ± 44.2	–
	10 C	32.9 ± 0.4	7.3 ± 0.53	0.9 ± 0.01	44.9 ± 0.4	1.1 ± 0.02	0.9 ± 0.01	1177 ± 101.6	–
	14 C	23.7 ± 0.1	11.6 ± 0.63	0.9 ± 0.01	30.4 ± 0.2	1.1 ± 0.01	0.9 ± 0.01	493.1 ± 35.1	–
	16 C	21.9 ± 0.3	11.9 ± 1.01	0.9 ± 0.01	29.1 ± 0.3	1.1 ± 0.01	0.9 ± 0.01	357.1 ± 32.6	–

**Table 3:** The comparative study of corrosion protection effect of LDHs/inhibitors on reinforcing bars.

Ref	Inhibitors/ Dosage	Media	NaCl mol/L	Test Method	Test Time (h)	*R_ct_*/*R_p_* (kΩ cm^2^)	Inhibition Efficiency (*η*%)
This paper	CaAl LDH-eugenol 1 g/L	simulated concrete pore solution	0.6	EIS	168	6118.1	99.9
This paper	CaAl LDH-eugenol 3 wt.%	mortar specimens	0.6	EIS	16 cycles	357.1	99.9
35	ZnAl LDH-NO_2_^−^ 25 g/L	simulated carbonated concrete pore solution	0.3	EIS	168	36.96	/
47	MgAl LDH-NO_2_^−^ 15 g/L	simulated carbonated concrete pore solution	0.3	EIS	216	960.8	99.84
48	ZnAl LDH-phthalates 20 g/L	simulated carbonated concrete pore solution	0.3	EIS	/	3.4	70.6
49	1,6-hexamethylenediamine 10 wt.%	mortar specimens	1	EIS	168	19.39	84.5

## Data Availability

The data presented in this study are available on request from the corresponding author.
